# Manic and hypomanic states in cancer patients: A systematic review

**DOI:** 10.1017/S1478951526102466

**Published:** 2026-05-08

**Authors:** Kyoko Osawa, Daisuke Fujisawa

**Affiliations:** 1 Department of Psychiatry and Palliative Care Center, Shiga General Hospital, Moriyama, Japan; 2 Department of Anesthesiology, Wakayama Medical University, Wakayama, Japan; 3 Department of Psychiatry, Graduate School of Medicine, Kyoto University, Kyoto, Japan; 4 Division of Quality Assurance Programs, National Cancer Center Institute for Cancer Control, Tokyo, Japan

**Keywords:** Mania, hypomania, cancer, bipolar disorder, depression

## Abstract

**Objectives:**

While there have been reports on the relationship between cancer and depression, reports on the association between cancer and manic states, a reciprocal state of depression, have been relatively few. Therefore, we conducted a systematic review on the relationships between cancer and manic states, focusing on their etiology, clinical course, and impact on cancer treatments.

**Methods:**

A systematic review was conducted using four electronic databases, following the PRISMA guidelines. The scope of the study included research on manic or hypomanic states associated with cancer in patients with no prior history of mental illness, published from 1950 to August, 2021. The study protocol was registered with PROSPERO (CRD42020182372).

**Results:**

Fifty-six studies, including 67 cases, were identified. The etiology of manic states in cancer patients was classified into organic, drug-induced, and psychogenic, with steroids being the most predominant causative agent. Approximately half of the patients discontinued cancer treatment following the onset of manic states. This was associated with a low rate of pharmacological treatment during the acute and maintenance phase of mania. The onset of manic states was most frequent during cancer treatment; however, about 15% of the cases exhibit manic symptoms before cancer diagnosis.

**Significance of results:**

This systematic review illustrated the clinical characteristics of manic state regarding differences in the etiology, timing of onset, pharmacological treatments, duration to remission, recurrence, and impact on cancer treatment. Manic states, which are comorbid with cancer, have significant clinical impacts on cancer prognosis. Therefore, appropriate pharmacological treatment for manic states is critical to consolidate appropriate cancer treatment. A substantial proportion of patients exhibit manic symptoms prior to the diagnosis of cancer, warranting further investigation into the possibility of the concept of “premonitory mania.”

## Introduction

There has been a considerable volume of literature addressing the intersection of cancer and mood disorders, particularly concerning the relationship between cancer and depression. It has been recognized as early as since the 1930s that depressive episodes may predispose an onset of cancer. Mood symptoms that emerge before cancer diagnosis have been known as “premonitory depression” (Lauter 1973; Cosci et al. 2015). A recent study demonstrated that 21% of patients with pancreatic cancer had a history of depression prior to their cancer diagnosis (Seoud et al. 2020; Michoglou et al. 2023). The argument has been continuing regarding whether depression arises as a psychological response to a cancer diagnosis, or a biological reaction to cancer. Recent research increasingly focuses on the biological mechanisms underlying the association between depression and cancer (Bortolato et al. 2017). Chronic exposure to elevated inflammatory cytokines due to cancer leads to alterations in neurotransmitter systems and can develop neuropsychiatric disorders such as depression (Felger and Lotrich 2013).

Bipolar disorder (BD) is a mental disorder that accompanies both depressive symptoms and manic symptoms. Its estimated prevalence rate is 2% and shows a gradual increase globally (Merikangas et al. 2011; He et al. 2020). In contrast with the research on depression and cancer, research on the relationship between BD or manic episodes and cancer has been relatively limited. Epidemiological data from large-scale cohort studies on breast cancer indicate that the incidence rate of breast cancer is 2.06 times higher in individuals with BD compared to the control group (Hung et al. 2013). Kanani et al. reported that breast cancer patients with comorbid mood disorders (depression or BD) experience poorer overall survival compared to those without such mood disorders (Kanani et al. 2016).

Literature to date suggests that the causes of manic states in patients with cancer have been categorized into three etiological perspectives: organic, drug-induced, and psychogenic. Krauthammer and Klerman (1978) introduced the concept of “secondary mania” in their seminal work on cancer and manic states. They reviewed various organic and pharmacological factors, such as drugs, infections, neoplasms, epilepsy, and metabolic disorders, that can induce manic states.

An increasing number of reports support the possibility of a pathophysiological link between cancer and BD (Asevedo et al. 2013). Cytokine-induced abnormalities in inflammation, hormones, and the immune system in individuals with cancer may represent a key pathophysiological pathway in the onset of manic episodes in BP (Goldberg and Schwertfeger 2010; Błogowski et al. 2014; Van den Ameele et al. 2016). Cytokine levels (such as TNF-α and IL-6), which rise in manic states, are known to increase in cancer patients as well. Hormonal abnormalities, specifically dysfunction of the hypothalamic-pituitary-adrenal axis, can cause impairments in neuroplasticity and brain dysfunction (Bauer et al. 2014; Rosenblat et al. 2014). The incidence of BP increases by 70% within five years following an autoimmune disease diagnosis (Eaton et al. 2010; Benros et al. 2014; León-Caballero et al. 2015).

Manic episodes may develop as a reaction to psychological stress (“psychogenic”) (Morgan et al. 2001), although the current diagnostic criteria, such as DSM-5 (American Psychiatric Association. 2013) and ICD-11 (World Health Organization 2019), do not formally recognize the term psychogenic mania. Studies have demonstrated the correlation between stressful life events and the onset or recurrence of manic episodes of BD (Kessing et al. 2004; Carmassi et al. 2020; Rodrigues Cordeiro et al. 2023).

BD remains a significant therapeutic challenge due to its symptom fluctuations and the resulting deterioration of patients’ functioning, heightened risk of suicide, and need for psychiatric hospitalization (Pompili et al. 2013; Harrison et al. 2018). Manic symptoms are particularly problematic due to patients’ heightened impulsiveness, aggression, irritability, and excitement (Gonçalves-Pinho et al. 2022). In cancer patients, manic episodes may hamper the initiation or continuation of cancer therapy due to the patients’ aggression and medication non-compliance. Typically, the treatment of psychiatric symptoms (including admission to psychiatric beds) is prioritized over cancer treatment, resulting in the postponement or cessation of cancer therapies, which can exacerbate the progression of cancer (Tohen and Grundy 1999). Additionally, patients with BD may refuse medication during their manic episodes, which also poses significant challenges for cancer treatment. Further, mistrust and aggression towards medical staff and family members prevent appropriate cancer treatment and care (Perlick et al. 1999; Osawa et al. 2013). Even after the manic episodes are remitted, the family may not be as emotionally supportive as before to the patient, which may lead to missed opportunity for the patient for appropriate cancer care. Also, psychological damage to family members may remain.

To the best of the authors’ knowledge, there are only sporadic studies of the emergence of manic states in the context of cancer, leaving a gap in understanding the etiology and the influence of manic episodes on cancer treatments. Therefore, in the current study, the authors conducted a systematic review to elucidate the characteristics of manic states in cancer patients. Once the review has been done, the authors expect that therapeutic strategies for manic episodes in patients with cancer would be made clearer.

## Method

We followed the Preferred Reporting Items for Systematic Reviews and Meta-Analyses (PRISMA) guideline (Page et al. 2021) and the protocol for this study was registered in the International Prospective Register of Systematic Reviews (PROSPERO) of the National Institute for Health and Care Research, UK (CRD42020182372). The following studies were eligible: (1) cases of comorbid cancer and manic states (manic or hypomanic states), (2) without a history of mental illness prior to cancer diagnosis, (3) epidemiological studies or case reports, and (4) written in English or Japanese. The following reports were excluded: (1) cases of benign proliferative diseases, or (2) cases with a history of mental illness that were not attributable to cancer. We searched the following electronic databases: PubMed, Cochrane, CINAHL, and Igaku Chuo Zasshi, for the studies published between 1950 and 6 August 2021. Boolean searches were performed using the following Mesh terms: (“mania” OR “hypomania” OR “bipolar disorder”) AND (“cancer”). After removing duplicates, two authors (K.O. and D.F.) screened the titles and abstracts, then reviewed the full texts. References from the selected papers were manually searched. Any discrepancies between the two reviewers were discussed until agreement.

**Figure 1.**
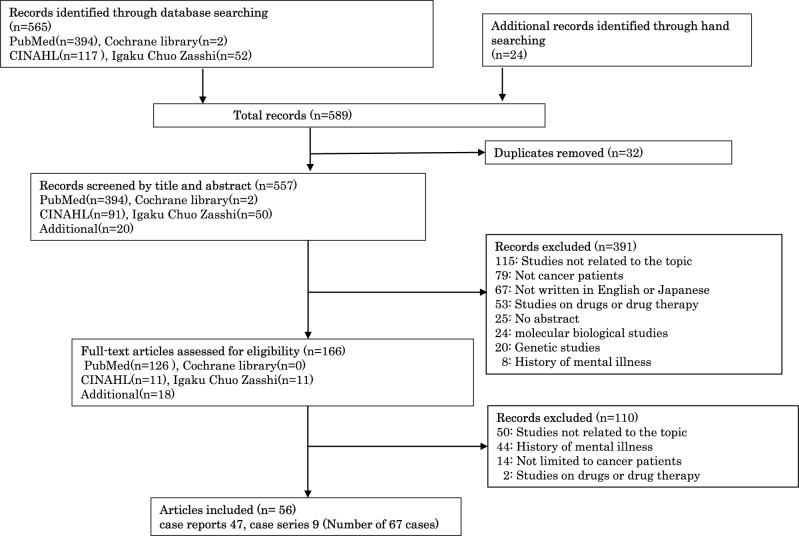
Flow diagram of the systematic literature search.

The following information was collected: the causes, timing of onset of manic states, their course and outcomes, including admissions to a psychiatric ward, pharmacotherapy and maintenance therapy for manic symptoms, duration until remission of manic states, recurrence of manic states, and the impact of manic states on cancer treatment. The flow diagram of the systematic literature search is presented in Figure 1.

## Results

The search finally retrieved 56 papers (9 case series and 47 case reports), which included 67 cases in total (see Table 1).

**Table 1.** Summary of reports on cancer patients presenting with manic state

No.	Author(s), year	Age	Sex	Type of Cancer	Cancer treatment status at the onset of mania	Underlying factors of mania
1	Shen and Lee 2021	65	M	Lung cancer	Before cancer diagnosis	Organic
2	El Hayek and El-Khoury 2020	57	M	ACTH-producing small cell lung carcinoma	Before cancer diagnosis	Organic and Drug-induced
3	Mavrotas and Murray 2020	66	F	Small cell lung cancer	During cancer treatment	Drug-induced
4	Garg et al. 2018	46	M	Carcinoma colon	During cancer treatment	Drug-induced
5	Schillinger 2017	75	M	Pancreas cancer	Before cancer diagnosis	Organic
6	Basterreche et al. 2017	66	M	Pancreatic adenocarcinoma	Before cancer diagnosis	Organic
7	Reich et al. 2017	61	F	Breast cancer	During cancer treatment	Organic and Drug-induced
8	Morosán Allo et al. 2015	62	F	Papillary thyroid carcinoma	During cancer treatment	Organic
9	Asevedo et al. 2013	39	F	Breast cancer	After cancer diagnosis – before cancer treatment	Organic
10	Brett et al. 2013	51	M	Skin cancer (squamous cell carcinoma)	During cancer treatment	Drug-induced
11	Olde Engberink et al. 2013	74	F	Large B-cell lymphoma	Before cancer diagnosis	Organic
12	Hechtman et al. 2013	14	M	T-cell acute lymphocytic leukemia	During cancer treatment	Drug-induced
13	Shirata et al. 2013	70	M	Gastric cancer	During cancer treatment	Psychogenic
14	Osawa et al. 2013	67	F	Pancreas cancer	During cancer treatment	Organic
15	Matsunaga et al. 2012	66	F	Gastric cancer	During cancer treatment	Psychogenic
16	Kimmel and Combs 2012	55	F	Breast cancer	During cancer treatment	Drug-induced
17	Cassidy et al. 2012	17	M	Acute lymphoblastic leukemia	During cancer treatment	Drug-induced
18	Roxanas and Hunt 2012	52	F	Multiple myeloma	unknown	Drug-induced
19	Roxanas and Hunt 2012	54	F	Multiple myeloma	unknown	Drug-induced
20	Roxanas and Hunt 2012	66	F	Multiple myeloma	unknown	Drug-induced
21	Roxanas and Hunt 2012	69	F	Breast cancer pulmonary infiltrates	unknown	Drug-induced
22	Roxanas and Hunt 2012	75	F	B cell lymphoma	unknown	Drug-induced
23	Tutkunkardas and Mukaddes 2010	14	M	T-cell acute lymphoblastic leukemia	During cancer treatment	Drug-induced
24	Ularntinon et al. 2010	8	M	T-cell acute lymphoblastic leukemia	During cancer treatment	Drug-induced
25	Ularntinon et al. 2010	16	M	Pre-B-cell acute lymphoblastic leukemia	During cancer treatment	Drug-induced
26	Enudi and Udolu 2009	30	M	Malignant melanoma	During cancer treatment	Drug-induced
27	Murru et al. 2009	91	M	Pancreas cancer	Before cancer diagnosis	Organic
28	Ni Mhaolain and Thakore 2007	54	M	Malignant melanoma	During cancer treatment	Drug-induced
29	Mercadante et al. 2007	48	M	myeloma IgG	During cancer treatment	Drug-induced
30	Mian et al. 2007	14	M	Hodgkin’s Lymphoma	During cancer treatment	Drug-induced
31	Hochhauser et al. 2005	15	M	B-lineage lymphoblastic lymphoma	During cancer treatment	Drug-induced
32	Hochhauser et al. 2005	6	M	Acute lymphoblastic leukemia	During cancer treatment	Drug-induced
33	Ingram and Hagemann 2003	2	M	Relapsed acute lymphoblastic leukemia	During cancer treatment	Drug-induced
34	Nakamura et al. 2003	74	M	Gastric cancer	During cancer treatment	Drug-induced
35	Greenberg et al. 2000	53	F	An acral lentiginous melanoma	During cancer treatment	Drug-induced
36	Greenberg et al. 2000	25	F	Melanoma	During cancer treatment	Drug-induced
37	Greenberg et al. 2000	20	M	Melanoma	During cancer treatment	Drug-induced
38	Onishi et al. 2000	66	M	Lung cancer	During cancer treatment	Psychogenic
39	Mazure et al. 1999	61	M	Glioblastoma	Before cancer diagnosis	Organic
40	Kramer and Cottingham 1999	14	F	Pre B-cell acute lymphoblastic leukemia	During cancer treatment	Drug-induced
41	Iancu et al. 1997	47	M	Chronic myeloid leukemia	During cancer treatment	Drug-induced
42	Strite et al. 1997	43	F	Chronic myelogenous leukemia	During cancer treatment	Drug-induced
43	Strite et al. 1997	47	M	Chronic myelogenous leukemia	During cancer treatment	Drug-induced
44	Morita et al. 1997	66	M	Gastric cancer	During palliative treatment	Psychogenic
45	Morita et al. 1997	79	Unknown	Tongue cancer	During palliative treatment	Drug-induced
46	Furutsuka et al. 1996	53	F	Renal cell cancer	During cancer treatment	Drug-induced
47	Sirois 1995	67	M	Unknown	During palliative treatment	Psychogenic
48	Watanabe et al. 1994	17	F	Acute lymphoblastic leukemia (ALL)	During cancer treatment	Drug-induced
49	Khouzam et al. 1994	64	M	Malignant melanoma	During cancer treatment	Drug-induced
50	Shinozaki et al. 1994	68	M	Breast cancer	During palliative treatment	Psychogenic
51	Murata, 1994	39	M	B cell lymphoma	Before cancer diagnosis	Organic
52	Stecker 1993	20	M	Osteogenic sarcoma	During palliative treatment	Psychogenic
53	Fukue et al. 1993	62	M	Hepatoma and laryngeal cancer	During palliative treatment	Psychogenic
54	Adams et al. 1988	52	M	Chronic myelogenous leukemia	During cancer treatment	Drug-induced and cognitive decline
55	Venkatarangam et al. 1988	68	M	Acute lymphoblastic leukemia (ALL)	During cancer treatment	Drug-induced
56	Collins and Oakley-Browne 1988	70	F	ACTH-secreting small cell carcinoma of the lung	Before cancer diagnosis	Organic
57	Starkstein et al. 1988	28	M	Astrocytoma	During cancer treatment	Organic
58	Motoyasu 1988	44	F	Pancreatic cancer	During palliative treatment	Psychogenic
59	Lajeunesse et al. 1986	75	M	Prostatic cancer	During cancer treatment	Drug-induced
60	Glynne-Jones et al. 1986	19	F	Hodgkin’s disease	During cancer treatment	Drug-induced
61	Glynne-Jones et al. 1986	56	M	Non-Hodgkin lymphoma	During cancer treatment	Drug-induced
62	Greenberg and Brown 1985	55	M	Lung cancer (middle lobe epidermoid carcinoma)	During cancer treatment	Organic
63	Wamboldt et al. 1984	25	F	Acute myelomonocytic leukemia	During cancer treatment	Drug-induced
64	Pickar et al. 1984	60	M	Adenocarcinoma of the pancreas	During cancer treatment	Drug-induced
65	Oyewumi and Lapierre 1981	21	M	Medulloblastoma	During cancer treatment	Organic
66	Jamieson and Wells 1979	45	M	Unknown primary cancer	Before cancer diagnosis	Organic
67	Mann and Hutchison 1967	38	M	Hodgkin’s disease	During cancer treatment	Drug-induced

### Overview of the cases

The overview of the 67 cases is shown in Table 2. The mean age of the cases was 47.9 years, with 64% of male patients. The most common cancer was leukemia (15 cases), followed by lymphoma (8 cases), lung cancer, pancreatic cancer, and malignant melanoma (6 cases each).

**Table 2.** Manic states in 67 cancer patients

	All cases (*n* = 67)	Drug-induced cases (*n* = 40)	Organic cases (*n* = 15)	Psychogenic cases (*n* = 9)
Average age (years)	47.9 ± 22.2	41.2 ± 23.0	57.2 ± 18.5	58.8 ± 16.5
Gender				
Male:	43 (64.%)	24 (60%)	10 (66.7%)	7 (77.8%)
Female:	23 (34.3%)	15 (37.5%)	5 (33.3%)	2 (22.2 %)
Not stated:	1 (1.5%)	1 (2.5%)	0 (0%)	0 (0 %)
**Common types of cancer**	1: Leukemia (15 cases)	1: Leukemia (14 cases)	1: Pancreatic cancer (4 cases)	1: Gastric cancer (3 cases)
	2: Lymphoma (8 cases)	2: Lymphoma (6 cases)	2: Lung cancer (3 cases)	
	3: Lung cancer (6 cases)	2: Malignant melanoma (6 cases)	3: Lymphoma (2 cases)	
	3: Pancreatic cancer (6 cases)	4: Myeloma (4 cases)		
	3: Malignant melanoma (6 cases)	5: Breast cancer (2 cases)		
**Cancer treatment status at the onset of mania**				
Before cancer diagnosis	10 (15.0%)	0 (0%)	9 (60%)	0 (0%)
After cancer diagnosis – before cancer treatment	1 (1.5 %)	0 (0%)	1 (6.7%)	0 (0%)
Under cancer treatment	44 (65.7%)	34 (85%)	5 (33.3%)	3 (33.3%)
Under palliative treatment (after active cancer treatment)	7 (10.4 %)	1 (2.5%)	0 (0%)	6 (66.7%)
Unknown	5 (7.5 %)	5 (12.5%)	0 (0%)	0 (0%)
**Acute drug treatment for mania**				
Yes:	52 (77.6%)	34 (85%)	11 (73.3%)	5 (55.6%)
None:	13 (19.4%)	6 (15%)	1 (6.7%)	4 (44.4%)
Not stated:	2 (1.5%)	0 (0%)	3 (20%)	0 (0%)
**Maintenance pharmacotherapy for mania**				
Yes:	34 (50.7%)	24 (60%)	6 (40%)	2 (22.2%)
None:	14 (20.9%)	7 (17.5%)	3 (20%)	4 (44.4%)
Not stated:	19 (28.4%)	9 (22.5%)	6 (40%)	3 (33.3%)
**Brain imaging**				
Yes:	37 (55.2%)	17 (42.5%)	14 (93.3%)	4 (44.4%)
No or not stated:	30 (44.8%)	23 (57.5%)	1 (6.7%)	5 (55.6%)
**Duration of manic state until remission (days)**				
Mean:	20.7	18.9	20.4	30.7
Median:	14	14	21	14
**Admission to psychiatric wards**				
Yes:	25 (37.3%)	11 (27.5%)	10 (66.7%)	3 (33.3%)
None:	23 (34.3%)	13 (32.5%)	2 (13.3%)	6 (66.7%)
Not stated:	19 (28.4%)	16 (40%)	3 (20%)	0 (0%)
**Continued or administration of cancer treatment after mania**				
Yes:	35 (52.2%)	22 (55%)	9 (60%)	2 (22.2%)
None:	13 (19.4%)	12 (30%)	2 (13.3%)	6 (66.6%)
Not stated:	19 (28.4%)	6 (15%)	4 (26.7%)	1 (11.1%)
**Relapse of manic episodes**				
Yes:	16 (23.9%)	12 (30%)	4 (26.7%)	0 (0%)
None:	44 (65.7%)	22 (55%)	10 (66.7%)	9 (100%)
Not stated:	7 (10.4%)	6 (15%)	1 (6.7%)	0 (0%)

### Etiology of manic episodes

The etiology of manic states was documented in 64 cases, which were classified into organic, drug-induced, and psychogenic. Drug-induced factors were most common (40 cases), followed by organic (15 cases), and psychogenic (9 cases). Three cases were attributed to the overlapping of drug-induced and organic factors. In Table 3, we presented the details of the organic cases.

**Table 3.** Details of organic cases

			Correspondence with the criteria by Krauthammer and Klerman (1978)				Association between the effectiveness of cancer treatment and the course of manic episodes
Author (s)	Publication year	Onset of manic episodes prior to cancer diagnosis	1. Proximity between an organic cause and the onset of manic episodes	2. Negative medical history prior to the onset of the manic state	3. Negative family history	4. Late-onset (65 years or older)	
Shen and Lee	2021	Yes	Two years before the cancer diagnosis	Yes	Not stated	Aged (65yo)	Yes
Schillinger	2017	Yes	Several months before the cancer diagnosis	Yes	Not stated	Aged (75yo)	Yes
Basterreche et al.	2017	Yes	Approximately four months before the cancer diagnosis	Yes	Not stated	Aged (66yo)	Unknown
Morosán Allo et al.	2015	No	Six weeks after surgery	Yes	Not stated	62yo	Yes
Asevedo et al.	2013	No	Two months after the cancer diagnosis	Yes	Yes	39yo	Unknown
Olde Engberink et al.	2013	Yes	Three weeks before the cancer diagnosis	Yes	Not stated	Aged (74yo)	No
Osawa et al.	2013	No	Three weeks after surgery	Yes	Yes	Aged (67yo)	Yes
Murru et al.	2009	Yes	Seven months before the cancer diagnosis	Yes	Not stated	Aged (91yo)	Unknown
Mazure et al.	1999	Yes	Several weeks before the cancer diagnosis	Yes	Not stated	61yo	Unknown
Murata	1994	Yes	One month before the cancer diagnosis	Yes	Yes	39yo	Yes
Collins and Oakley-Browne	1988	Yes	About 10 days before the cancer diagnosis	Yes	No	Aged (70yo)	Unknown
Starkstein et al.	1988	No	After recurrence and undergoing radiation therapy	Yes	Yes	28yo	No
Greenberg and Brown	1985	No	Seven months after lung cancer treatment, a brainstem metastasis developed, leading to the onset of a manic state. Five months later, when the manic state recurred, the brain metastasis also expanded.	Yes	Yes	55yo	Yes
Oyewumi and Lapierre	1981	No	After surgery and radiation therapy	Yes	Not stated	21yo	Unknown
Jamieson and Wells	1979	Yes	Four months before the cancer diagnosis	Yes	No	45yo	Unknown

### Timing of the onset of manic episodes

Manic states predominantly occurred during cancer treatment (44 cases: 66%), followed by those that occurred before cancer diagnosis (10 cases: 15%). Of the 10 cases that exhibited the onset of manic episodes prior to the diagnosis of cancer, contributing factors for the manic episodes were identified in nine cases.

### Clinical management of manic episodes

Pharmacotherapy for manic states was administered in 52 cases (78%). Maintenance pharmacotherapy after remission of manic states was administered in 34 cases (51%). The administered pharmacotherapy for manic states is summarized in Table 4. Among the 52 cases where pharmacotherapy was administered, monotherapy with an antipsychotic or a mood stabilizer was most common, comprising 22 cases (40%). Combinations of antipsychotics and mood stabilizers, often accompanied by benzodiazepines, were also common. While benzodiazepines were frequently used for sleep, their daytime administration was also noted. In challenging cases, the first antipsychotic was switched to another or was augmented with other classes of medications.

**Table 4.** Medications used to treat manic states

	Number of cases	Maximum daily use (mg)	Chlorpromazine equivalent (mg)	Diazepam equivalent (mg)
**Antipsychotics**				
Haloperidol	13	1.5–12	75–600	
Risperidone	9	0.5–5	50–500	
Olanzapine	8	5–20	200–800	
**Mood stabilizers**				
Valproic acid	11	300–1000		
Lithium	10	300–600		
**Benzodiazepines**				
Lorazepam	6	1–4		10–40
Clonazepam	4	0.5–2		10–40

Multiple medications were used in some cases; therefore, the total number of cases in the table outweighs the actual number of cases.

Among the antipsychotics, haloperidol was predominantly used, followed by risperidone and olanzapine. Among mood stabilizers, both valproic acid and lithium were frequently used. In the cases of drug-induced manic states, reducing or discontinuing causative medications such as steroids or interferon while concurrently administering therapeutic agents for the manic state was often observed. The doses of medications were generally comparable to or lower than those used for common mood disorders (Marzani and Neff 2021).

Brain imaging was conducted in 37 cases (55%).

### Clinical course of manic episodes and cancer treatment

The mean and median duration until remission of manic states were 21 and 14 days, respectively. Approximately one third of the cases required hospitalization to psychiatric wards (25 cases: 37%).

Cancer treatment was continued or initiated after the onset of manic states in 35 cases (52%). In a subset of 34 cases with complete relevant data, administration of maintenance pharmacotherapy, not in the palliative phase, and drug-induced or organic etiologies were significantly associated with continuation of cancer treatment. Administration of pharmacotherapy for acute-phase manic states showed a trend-level association with continued cancer treatment (Table 5). The recurrence of manic states was observed in 16 cases (24%).

**Table 5.** Comparison of cases who did and did not receive cancer treatment after a manic state

		Cases who received cancer treatment	Cases who did not receive cancer treatment	*p*-value
		*n* = 20	*n* = 14	
Acute pharmacotherapy for mania				0.061†
	Yes	20 (64.5%)	11 (35.5%)	
	No	0(0.0%)	3(100.0%)	
Maintenance pharmacotherapy for mania				0.012*
	Yes	19 (70.4%)	8 (29.6%)	
	No	1 (14.3%)	6 (85.7%)	
Cancer treatment status at the onset of mania				0.010*
	Before cancer treatment	5 (83.3%)	1 (16.7%)	
	Under cancer treatment	15(65.2%)	8 (34.8%)	
	Under palliative treatment	0 (0.0%)	5 (100.0%)	
Relapse of manic episodes				0.704
	Yes	6 (66.7%)	3 (33.3%)	
	No	14 (56.0%)	11 (44.0%)	
Admission to psychiatric wards				0.728
	Yes	12 (20.0%)	7 (80.0%)	
	No	8 (53.3%)	7 (46.7%)	
Underlying factors of mania				0.030*
	Drug-induced	12 (60.0%)	8 (40.0%)	
	Organic	7 (87.5%)	1 (12.5%)	
	Psychogenic	1 (16.7%)	5 (83.3%)	

* *P* < 0.05, ^†^*P* < 0.10

## Discussion

To the best of the authors’ knowledge, this is the first systematic review that investigated manic states in cancer patients and their influence on cancer treatment.

### Clinical characteristics of cancer patients presenting with manic states

The reported cases of cancer patients who developed manic episodes tended to be relatively young, predominantly male, and with hematological malignancies. The manic states most frequently occurred during cancer treatments; however, a substantial proportion of patients (15%) exhibited manic symptoms preceding the cancer diagnosis.

Only half of the cases were able to continue or commence cancer treatment, elucidating the substantial adverse impact of manic episodes on cancer therapy. Nearly 40% required admission to psychiatric wards, indicating the severity of their manic symptoms.

### Etiology of manic episodes and their characteristics

Among the three categories of etiology of manic episodes, drug-induced manic states were the most frequent etiology, composing 60% of the cases. Steroid, which has been widely known a causative agent (Ismail et al. 2017), was the most common.

Organic cases were relatively few; however, since brain imaging was not performed in approximately half of the cases, there are chances of overlooked cases.

We compared the clinical characteristics of the three categories. As shown in Table 2, drug-induced cases were relatively young (at 40s), compared with organic and psychogenic cases (late 50s). This can be attributed to the high incidence of leukemia cases in the drug-induced category, which frequently requires high-dose steroids.

A substantial proportion of the patients experienced recurrence of manic episodes in drug-induced and organic cases, while there was no case of recurrence in psychogenic cases. Psychogenic cases were more likely to be seen among patients at the terminal stage, where many patients deceased early in their clinical course.

### Timing of the onset of manic episodes and their characteristics

The timing of the onset of manic states varied according to the causative factors: 85% of drug-induced cases occurred during cancer treatment, while many organic cases (60%) presented before the cancer diagnosis, and many psychogenic cases (67%) arose during palliative care.

A substantial proportion (15%) of patients exhibited manic episodes before the diagnosis of cancer. The mean age of onset for mania was 65, which is relatively higher than that of BD in general (Merikangas et al. 2011; Kroon et al. 2013). A few sporadic cases of manic episodes that preceded the diagnosis of pancreatic cancer (Pereira et al. 2022) have been reported. Sami et al. argued that the first hypomanic episode that occurred after the age of 50 is suggestive of organic factors (Sami et al. 2015).

In the current review, we collected the reports in which the authors of the report argued that patients’ manic state was associated with cancer; however, many reports lacked solid evidence to support their arguments. As shown in Table 3, none of the reported cases satisfied the criteria of Krauthammer & Klerman. Further, in their criteria, no specific threshold has been proposed regarding the proximity between an organic cause and the onset of manic episodes.

It has long been known that depressive episodes can precede a diagnosis of cancer, which is known as “premonitory depression” (Lauter 1973). In terms of manic episodes, evidence does not appear sufficient to support a clinical entity of concept of “premonitory mania,” despite there are sporadic reports of manic episodes that precede a cancer diagnosis. Further investigation into the possibility of the concept of “premonitory mania” is warranted.

### Clinical management of manic episodes

According to the practice guidelines for BD, pharmacotherapy is the standard approach for manic states (Tajika et al. 2022). Significant relief of manic symptoms is usually seen within two weeks (Li et al. 2017). Given the high relapse rate of BD (Keller et al. 1993), maintenance pharmacotherapy is recommended after remission of manic episodes (Kishi et al. 2022). However, in the current review, approximately 20% of the cases with manic episodes did not receive pharmacotherapy, and approximately half of the patients did not receive maintenance pharmacotherapy. The high rate of disrupted cancer therapy may be partly explained by the low rate of administering pharmacotherapy for the acute and maintenance phases of BD. No significant adverse effects have been reported in the use of mood stabilizers and/or antipsychotics in treating manic episodes. There was variability in the dosages of mood stabilizers, and even small doses were effective in many cases. Considering that the median duration to remission of manic states was as short as 14 days even in cancer patients, increasing the awareness of manic states could lead to better management that lead to better cancer treatment.

The current study revealed that brain imaging was conducted in only 55% of the cases. Since central nervous system (CNS) involvement of cancer, such as brain metastases and leptomeningeal metastases, can develop psychiatric symptoms, and the course of manic episodes differs depending on the underlying causes, CNS assessments, such as brain imaging, lumbar puncture, and electroencephalography, are recommended for cancer patients who exhibit psychiatric symptoms.

### Clinical course of manic episodes and cancer treatment

After the onset of manic episodes, cancer treatment was provided in 55% of drug-induced cases and 60% of organic cases, but only in 22% of psychogenic cases, suggesting that manic episodes had a significant impact on cancer treatment.

The overall duration until the remission of manic states in drug-induced and organic cases was similar to that of patients in general psychiatric practice (Gonzalez-Pinto et al. 2010). The median duration until the remission of manic states is longer in organic causes than in drug-induced cases, suggesting that organic cases are likely to present with more likely to be treatment-resistant. The duration until remission was relatively long in psychogenic cases, which is attributable to the fact that psychogenic cases occurred in patients in the palliative-care phase, presenting with milder manic symptoms; therefore, no intense psychopharmacological treatment was provided to such patients.

## Limitations and implications

The current review has a few limitations. First, this is a narrative review of cancer patients who had manic episodes. The association of manic episodes and cancer relied on the judgment of the reporting authors, and the evidence for the relationship between cancer and manic episodes has not been verified. Second, the classification of contributing factors to the onset of manic episodes (drug-induced, organic, and psychogenic) was based on the argument by the reporting authors. Further, these categories do not match the latest classification of mental illnesses, such as DSM-5 and ICD-11; therefore, the validity of the clinical entity is not established.

Despite these limitations, the current review is worth noting, since, to the best of the authors’ knowledge, it is the first comprehensive review of cancer and manic episodes. Further epidemiological and pathophysiological research is warranted to better understand the linkage between cancer and manic episodes.

## Supporting information

10.1017/S1478951526102466.sm001 Osawa and Fujisawa supplementary material 1 Osawa and Fujisawa supplementary material

10.1017/S1478951526102466.sm002 Osawa and Fujisawa supplementary material 2 Osawa and Fujisawa supplementary material

## Supplementary material

The supplementary material for this article can be found at https://doi.org/10.1017/S1478951526102466.
