# Manic and hypomanic states in cancer patients: A systematic review – ERRATUM

**DOI:** 10.1017/S147895152610296X

**Published:** 2026-07-06

**Authors:** Kyoko Osawa, Daisuke Fujisawa

The Publisher apologises for an incorrect file being published as the Supplementary Material for this article. This has now been removed and replaced with the correct file.
